# Morphological and Spatial Heterogeneity of Microbial Communities in Pilot-Scale Autotrophic Integrated Fixed-Film Activated Sludge System Treating Coal to Ethylene Glycol Wastewater

**DOI:** 10.3389/fmicb.2022.927650

**Published:** 2022-06-02

**Authors:** Fangxu Jia, Jiayi Chen, Xingcheng Zhao, Chenyu Liu, Yiran Li, Jinyuan Ma, Anming Yang, Hong Yao

**Affiliations:** Beijing International Scientific and Technological Cooperation Base of Water Pollution Control Techniques for Antibiotics and Resistance Genes, Beijing Key Laboratory of Aqueous Typical Pollutants Control and Water Quality Safeguard, School of Environment, Beijing Jiaotong University, Beijing, China

**Keywords:** anammox, the integrated fixed-film activated sludge (IFAS), partial nitritation/anammox (PN/A), heterogeneity, population shifts

## Abstract

The understanding of microbial compositions in different dimensions is essential to achieve the successful design and operation of the partial nitritation/anammox (PN/A) process. This study investigated the microbial communities of different sludge morphologies and spatial distribution in the one-stage PN/A process of treating real coal to ethylene glycol (CtEG) wastewater at a pilot-scale integrated fixed-film activated sludge (IFAS) reactor. The results showed that ammonia-oxidizing bacteria (AOB) was mainly distributed in flocs (13.56 ± 3.16%), whereas anammox bacteria (AnAOB) was dominated in the biofilms (17.88 ± 8.05%). Furthermore, the dominant AnAOB genus in biofilms among the first three chambers was *Candidatus Brocadia* (6.46 ± 2.14% to 11.82 ± 6.33%), whereas it was unexpectedly transformed to *Candidatus Kuenenia* (9.47 ± 1.70%) and *Candidatus Anammoxoglobus* (8.56 ± 4.69%) in the last chamber. This demonstrated that the niche differentiation resulting from morphological (dissolved oxygen) and spatial heterogeneity (gradient distribution of nutrients and toxins) was the main reason for dominant bacterial distribution. Overall, this study presents more comprehensive information on the heterogeneous distribution and transformation of communities in PN/A processes, providing a theoretical basis for targeted culture and selection of microbial communities in practical engineering.

## Introduction

The anaerobic ammonia oxidation (anammox) was recognized as an efficient and sustainable alternative to the conventional biological denitrification process due to its advantages of no organic carbon required and aeration savings (Ma et al., 2016). Among the many anammox coupled nitrogen removal processes, one-stage partial nitritation/anammox (PN/A) was greatly favored due to the more efficient and low cost (Lackner et al., 2014). Nevertheless, the long-term stable operation of the one-stage PN/A process still presents two major challenges: the balance of cooperation between anammox bacteria (AnAOB) and ammonia-oxidizing bacteria (AOB) and suppressing the growth of nitrite-oxidizing bacteria (NOB) (Zheng et al., 2016).

The integrated fixed-film activated sludge (IFAS) reactor served as an emerging technology that can be used to solve the above problems. It combined two types of sludge (biofilm and flocs) in a single system, which provided the optimal habitat for AnAOB and AOB cooperation, meanwhile inhibiting the growth of NOB by flocs sludge discharge (Zhang et al., 2022). Therefore, an increasing number of studies have begun to focus on the operation mode and wastewater treatment efficiency of IFAS, while the research on microbial community dynamics, especially for AnAOB population transformation in plug-flow mode, was limited. Due to differences in physiological characteristics of AnAOB at the genus level, the niche differentiation among genera likely occurred during the change in environmental conditions. For example, *Candidatus Brocadia* (*K_NO2–_* = 34–350 μM) preferred to enrich in high nitrite concentrations than *Candidatus Kuenenia* (*K_NO2–_* = 0.2–3 μM) (Zhang and Okabe, 2020); *C. Kuenenia* (*K_o2_* = 0–200 μM) was able to survive in suspended sludge due to the higher oxygen tolerance compared with *C. Brocadia* (*K_o2_* = 1–120 μM) and *Candidatus Scalindua* (*K_o2_* = 10–20 μM) (Oshiki et al., 2016); *C. Brocadia* and *Candidatus Anammoxoglobus* can survive in the presence of organic matter due to their metabolic diversity (Kartal et al., 2007a,b). These studies suggested that different AnAOB genera may present interspecific competition based on differences in survival space and substrate concentration, resulting in the elimination or reduced abundance of inferior species in the habitat.

Currently, many studies have reported population shifts among various AnAOB genera under environmental and operating condition changes in upflow and completely mixed-mode reactors. For example, *C. Brocadia* in both suspended and biofilm sludge shifted to *C. Kuenenia* with the gradual reduction of hydraulic retention time in a UASB reactor (Park et al., 2015), and the original dominant *C. Brocadia* was shifted to *C. Anammoxoglobus* after increasing the propionic acid concentration in an SBR reactor (Kartal et al., 2007b) or gradually reducing temperature and ammonia in the CANON reactor (Gonzalez-Martinez et al., 2016). However, the above studies only tended to concentrate on revealing the relationship between AnAOB population shifts and surrounding environments or operations within the overall reactor, and less attention was paid to the morphological and spatial heterogeneity of the AnAOB genus at different zones in the plug-flow mode IFAS reactor. Furthermore, the IFAS reactor with a plug-flow mode is most likely to develop the population shift of AnAOB genera during long-term operation due to the degradation of pollutants along the flow direction, especially in the pilot- or full-scale IFAS for real industrial wastewater treatment.

Therefore, this study applied a pilot-scale IFAS reactor for the treatment of real coal to ethylene glycol (CtEG) wastewater and used 16S rRNA high-throughput sequencing and various biometrical methods to investigate the following two objectives: (1) the distribution of functional bacterial populations in sludge with different morphologies and spatial locations and (2) the potential factors influencing the transformation of dominant anammox genus. The results could provide a reference for the community composition and transformation of the PN/A process, which is essential to achieve IFAS systems efficiently treating similar types of wastewater in full-scale engineering applications.

## Materials and Methods

### Reactor Construction and Wastewater

**FIGURE 1:**
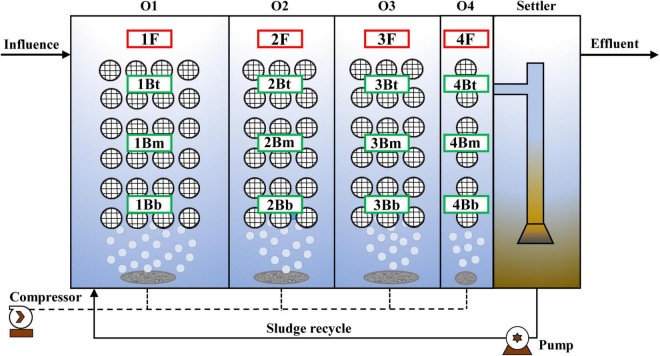
Schematic diagram of the pilot-scale integrated fixed-film activated sludge (IFAS) reactor and sampling scheme.

A simplified schematic of the pilot-scale IFAS reactor is illustrated in Figure 1. The effective volume of IFAS was 2 m^3^ (volume ratio was 3:2:2:1 for each chamber) and operated under oxic phase (DO < 0.5 mg⋅L^–1^) for treatment of CtEG wastewater originating from the full-scale plant in Tongliao City, Inner Mongolia, China. The sponge carriers with mature anammox biofilms (dominated by *C. Brocadia*, Supplementary Figure 1) were used as seed sludge for the anammox reactor start-up. Additionally, the polyurethane sponge carriers (size of 10 × 10 × 10 mm, density of 0.9 g⋅cm^–3^, and specific surface area of 2,800 m^2^⋅m^–3^) were fixed in the reactor with a special design as follows: the sponge carriers were first filled into spherical cages and connected together with steel wires, and then the steel wires were fixed to the shelves (Supplementary Figure 2). In addition, the detail of the operational procedure and the wastewater characteristics were as previously reported (Yao et al., 2021).

### Sampling and Microbial Community Analysis

Sludge samples were collected in triplicate from flocculent and biofilm sludge in each chamber at 132 days of the stabilization phase of the reactor. The sample names are as shown in Figure 1 where F represents the floc, B represents the biofilm, t, m, and b represent the top, middle, and bottom, respectively, and the numbers 1–4 indicate the chamber numbers. Therefore, a total of 12 (4 × 3) flocculent sludge samples and 36 (12 × 3) biofilm samples were obtained.

Genomic DNA was extracted from each sample using the FastDNA SPIN Kit for Soil (QBIOgene Inc., Carlsbad, CA, United States). The 16S rDNA V3-V4 region was specifically amplified with bacterial universal primers 338F (5′-ACTCCTACGGGAGGCAGCAG-3′) and 806R (5′-GGACTACHVGGGTWTCTAAT-3′). Subsequently, the PCR products were purified using the AxyPrep DNA Gel Extraction Kit (Axygen Biosciences, Union City, CA, United States). Amplification products of all samples were sequenced on the Illumina MiSeq PE300 platform. Sequences shorter than 200 bps, homopolymers with 6 bps and primer mismatches, as well as quality scores below 25 and chimeras were removed. High-quality sequences were treated with the Mothur software program (version 1.44.3) using a 97% sequence similarity threshold to generate operational taxonomic units (OTUs). Additionally, subsampling was performed to an equal sequencing depth of 24,338 reads per sample using the “single_rarefaction.py” module of the QIIME software (version 1.9.1). The raw sequencing data were uploaded to the NCBI Sequence Read Archive under accession no. PRJNA795428.

### Spectral and Chemical Analytical Methods

Wastewater samples were collected in triplicate from each chamber at 132 days. The excitation-emission matrix (EEM) fluorescence spectroscopy protocol was modified according to previous research (Jia et al., 2016). In brief, the samples were diluted with deionized water to eliminate the inner-filter effect of fluorescence. The EEM fluorescence spectroscopy was measured using luminescence spectrometry (F-7000, Hitachi, Japan). The spectrometer displayed a maximum emission intensity of 5,000 arbitrary units (AUs). The EEM spectra were collected with subsequent scanning emission spectra from 290 to 550 nm by varying the excitation wavelength from 200 to 400 nm. To eliminate second-order Raleigh scattering, a 290-nm cutoff was used in scanning. The spectrum of deionized water was recorded as a blank.

The concentrations of COD, NH_4_^+^-N, NO_2_^–^-N, and NO_3_^–^-N were determined according to standard methods (APHA, 2005). Furthermore, pH, DO, and temperature were monitored with WTW-pH/Oxi 340i oxygen/pH probes (WTW Company, Germany). The chemical analyses were performed in duplicate using analytical grade chemicals.

### Statistical Analysis

Principal component analysis (PCA) with Adonis pairwise test was performed using R (Version 4.1.0). The significantly different taxa in the microbial communities of each sample group were identified using the linear discriminant analysis (LDA) effect size (LEfSe) method.^1^ In addition, the Welch’s *t*-test was used to calculate statistical differences between core functional microorganisms in different groups using the SPSS software (version 26.0).

## Results

### The Performance of the Partial Nitritation/Anammox System

**FIGURE 2:**
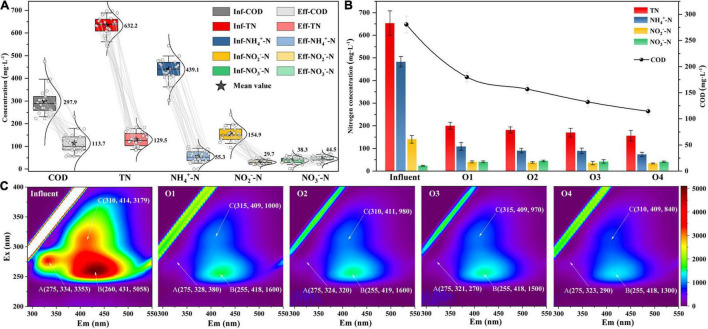
**(A)** The pollutant removal performance of IFAS reactor during stable operation stage, **(B)** pollutant concentration, and **(C)** fluorescence components along the plug-flow direction in each chamber.

The variation of pollutant concentration in the IFAS reactor during the stable operation stage (123–145 days) is shown in Figure 2A. During this stage, the nitrogen removal rate (NRR) was 0.5 ± 0.1 kg N⋅m^–3^⋅day^–1^, the COD removal rate was 62.0 ± 11.5%, and the total nitrogen removal efficiency (NRE) was 79.6 ± 4.4%. Furthermore, the values of the ratio between nitrate production and ammonium consumption (1.5 ± 2.3%) were obviously lower than the theoretical value of 11% (Miao et al., 2016), which were confirmed to be associated with the NO_3_^–^-N reduction process by denitrification (Yao et al., 2021). In addition, the evolution of the pollutant concentration in each chamber of the reactor was also analyzed on day 132 (Figure 2B). To be specific, the largest contribution to NRE was achieved by the first chamber (69.3 ± 7.3%) and gradually improved to 76.1 ± 2.1% in the last chamber. Meanwhile, the COD removal rates showed a similar pattern along the plug-flow direction (35.8 ± 3.8% to 59.1 ± 2.9%).

To further understand the evolution of organic substance components in the wastewater of the PN/A system, EEM fluorescence spectral analysis was carried out (Figure 2C). According to the division principle of fluorescence spectral regions (Chen et al., 2003), three fluorescence peaks were identified from EEM fluorescence spectra of the wastewater samples in all chambers that can be attributed to soluble microbial by-product (SMP)-like (peak A, *E*_x_/*E*_m_ = 275/321–334), the fulvic acid-like (peak B, *E*_x_/*E*_m_ = 255–260/418–431), and humic acid-like (peak C, *E*_x_/*E*_m_ = 310–315/409–414) component. Among the three peaks, peak B had the highest fluorescence intensity in influent and subsequently decreased from 68.4% (chamber 1) to 18.8% (chamber 4) with plug flow. The regularity of fluorescence intensity distributions of peak C (68.5–16.0%) coincided with that of peak B, whereas almost all the SMP-like substances (peak A) were degraded in the first chamber (88.7%) and virtually undetectable in the subsequent chambers. These results may be related to the extent of biodegradable organic matter, that is, humic acid and fulvic acid-like substances were refractory organics (Fang et al., 2019).

### Microbial Community Composition

The 16S rRNA high-throughput sequencing was used to investigate the microbial communities in different chambers for floc and biofilm samples, which were collected at 132 days of the stable operation period in the IFAS reactor. The α-diversity parameters of the microbial communities in all samples are concluded in Supplementary Table 1. The Good’s coverage of all OTUs was higher than 98.9%, and the rarefaction curves also showed clear and smooth horizontal asymptotes (Supplementary Figure 3), and both results suggest a near-complete sampling of the community. As for the Chao 1 index (Supplementary Figure 4A), group B (biofilm samples) was significantly higher than that of group F (floc samples) (*p* < 0.001), which indicated that the biomass abundance on the biofilm was higher than that of the flocculent sludge. In addition, the Chao 1 index of biofilm samples in each chamber displayed an obvious decreasing trend with flow direction, and the biofilm samples in chamber 1 (group 1B) was significantly higher than others (*p* < 0.05), which may be due to the higher nutrients in the front chamber that favors microbial colonization. In contrast to the Chao 1 index, the Shannon index of group F was significantly higher than that of group B (*p* < 0.001) and no significant difference between biofilm samples in each chamber (Supplementary Figure 4B). Apart from the above, there was no significant difference in the index of floc samples from each chamber and biofilm samples from different depths.

**FIGURE 3:**
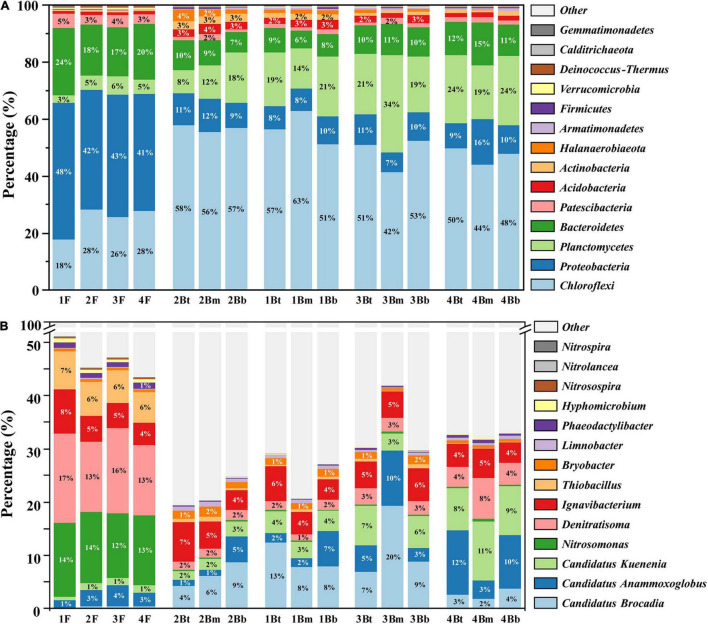
Microbial community composition in each sample. The relative abundance at the phylum level **(A)** and genus level **(B)**.

The microbial community structures of floc and biofilm samples from different chambers are depicted in Figure 3. At the phylum level, the predominant phyla for both floc and biofilm samples were *Chloroflexi*, *Proteobacteria, Planctomycetes*, and *Bacteroidetes*, which together accounted for more than 85% of the total microbial community detected. Obviously, the abundance of bacterial phyla detected in the two groups was dramatically different. Specifically, *Proteobacteria* and *Bacteroidetes* were dominant in the flocculent sludge, while *Chloroflexi* and *Planctomycetes* occupied higher proportions in the biofilm sludge. At the genus level, the dominant genera of flocculent sludge were *Denitratisoma* (14.8 ± 1.6%), *Nitrosomonas* (13.2 ± 0.7%), and *Thiobacillus* (6.4 ± 0.5%). While in the biofilm sludge, the dominant genera were three types of AnAOB, namely, *C. Brocadia* (7.6 ± 5.0%), *C. Kuenenia* (5.2 ± 3.3%), and *C. Anammoxoglobus* (5.1 ± 4.2%). In addition, *Ignavibacterium*, as a type of fermentative bacterium, was dominantly observed in both flocculent (5.5 ± 1.7%) and biofilm (5.1 ± 1.1%) sludge.

**FIGURE 4:**
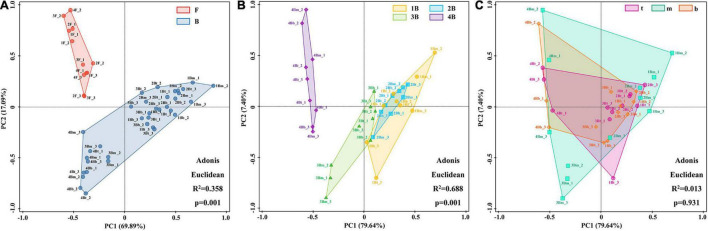
Principal component analysis (PCA) of bacterial population distribution in different groups at the genus level. **(A)** Flocs vs. biofilms; **(B)** biofilms in each chamber; and **(C)** biofilms in different depths.

Additionally, the PCA was used to distinguish sample cluster distribution in morphological and spatial dimensions. As shown in Figure 4A, floc and biofilm samples were clustered according to their respective sludge morphology and showed clear separation (*p* = 0.001). Meanwhile, an obvious grouping of the biofilm samples in each chamber was observed (*p* = 0.001) (Figure 4B). In addition, the grouping trends (chambers 1–4) were distinct shifts along PC1 axes from right to left, although there was a partial overlap between groups 1B and 2B. However, the samples at different depths of each chamber could not be separated effectively and showed large overlapping areas with each other (*p* = 0.931) (Figure 4C). The above results indicated that the difference in microbial community composition was significant in different morphologies and chambers, while not significant in different depths.

**FIGURE 5:**
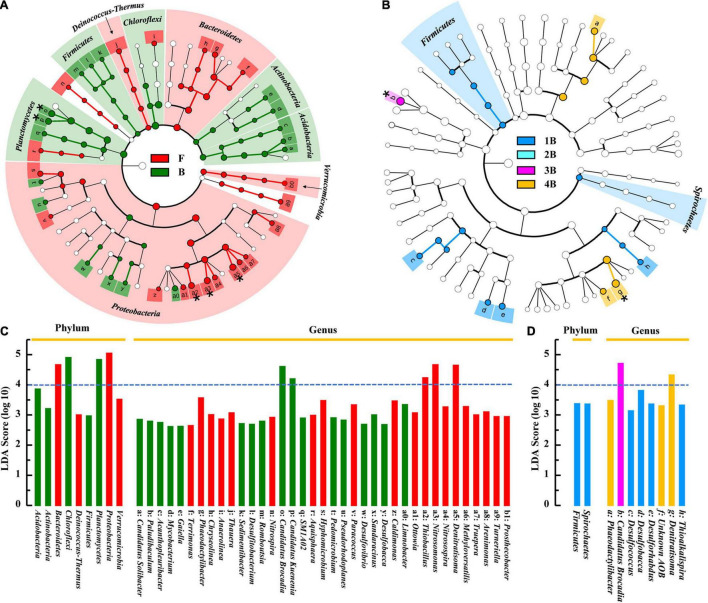
Differential analysis of microbial communities in different groups of IFAS reactor by LEfSe. Phylogenetic dendrogram of biomarker bacteria in floc and biofilm samples **(A)** and in biofilm samples of each chamber **(B)**; the LDA score of the abundant biomarkers in floc and biofilm samples **(C)**, and in biofilm samples of each chamber **(D)**. The linear discriminant analysis (LDA) score larger than 4 was marked with an asterisk in the phylogenetic dendrogram.

The significantly different taxa of sludge samples in different groups were further analyzed by the LEfSe algorithm (Figure 5). By comparing the F and B groups (Figures 5A,C), it was found that the most representative biomarkers (LDA score > 4) in flocculent were *Proteobacteria* (contain *Nitrosomonas*, *Denitratisoma*, and *Thiobacillus* members) and *Bacteroidetes*, while in biofilm sludge were *Chloroflexi* and *Planctomycetes* (contain *C. Brocadia* and *C. Kuenenia* members). These statistical results were basically consistent with the visual differences between the F and B groups (see above). As for biofilm samples in each chamber, the number of differential microorganisms decreased markedly compared with the F-B group (Figures 5B,D). The *Firmicutes* and *Spirochetes* were filtered as biomarkers in group 1B at the phylum level, even with lower LDA scores, whereas *C. Brocadia* and *Denitratisoma* were the most representative biomarkers in groups 3B and 4B at the genus level, respectively. As expected, there were no significantly different taxa in biofilm samples from different depths (data not shown), which showed the same results as the α-diversity.

### Core Functional Microorganism

**FIGURE 6:**
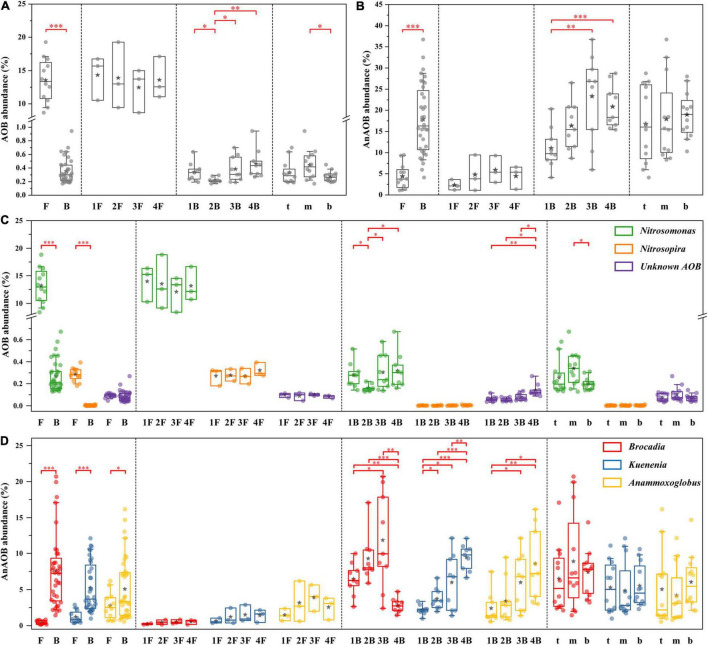
Distribution of core functional microorganisms in different groups. The relative abundance of overall ammonia oxidizing bacteria (AOB) **(A)** and anammox bacteria (AnAOB) **(B)**; the relative abundance of AOB **(C)** and AnAOB **(D)** at genus level. Welch’s *t*-test, asterisk (*) indicates *p* < 0.05, asterisk (^**^) indicates *p* < 0.01, asterisk (^***^) indicates *p* < 0.001.

The distribution characteristics of core functional microorganisms of the PN/A process in flocculent and biofilm sludge from each chamber were compared (Figure 6). This system detected three types of AOB (*Nitrosomonas*, *Nitrosospira*, and *Unknown AOB*) and three types of AnAOB (*C. Brocadia*, *C. Kuenenia*, and *C. Anammoxoglobus*). As shown in Figures 6A,C, AOB was mainly concentrated in flocculent sludge (13.6 ± 3.2%), where *Nitrosomonas* was the dominant genus with 97% of the total AOB. Other genera of AOB exhibited an identical distribution pattern with *Nitrosomonas*, but the abundance was extremely low and can be ignored. In contrast, the AnAOB was mainly enriched in biofilm (17.9 ± 8.1%), and only a small amount of *C. Kuenenia* (1.2 ± 0.8%) and *C. Anammoxoglobus* (2.8 ± 1.8%) were detected in flocculent sludge with almost no detection of *C. Brocadia* (0.4 ± 0.3%) (Figures 6B,D). Among the biofilms of each chamber, the content of *C. Brocadia* increased from 6.5 ± 2.1% to 11.8 ± 6.3% in the first three chambers and decreased substantially to 2.8 ± 1.0% in the last chamber. Nevertheless, the abundance of *C. Kuenenia* and *C. Anammoxoglobus* gradually increased along with the plug-flow direction from 2.2 ± 0.6% to 9.5 ± 1.7% and 2.4 ± 2.1% to 8.6 ± 4.7%, respectively. Moreover, almost no significant differences in core functional microorganisms could be observed either between flocculent sludge in different chambers or between biofilm sludge in different depths. Besides, the abundance of NOB was extremely low in both flocculent (0.3 ± 0.02%) and biofilm sludge (0.03 ± 0.01%), thus it will not be discussed in this study.

## Discussion

### Morphological Heterogeneity of Flocs and Biofilms

Balancing the cooperation between AOB and AnAOB for nitrite was key to the successful operation of the PN/A process. This study effectively solves this problem by integrating flocs and biofilms to separate functional bacteria in different spaces. As shown in Figures 4, 6, AOB preferred to grow in flocculent sludge (13.6 ± 3.2%), while AnAOB was mainly distributed in biofilms (17.9 ± 8.1%). Previous studies indicated that the morphological heterogeneity of microbial community was determined by the differences in the characteristic of the floc and the biofilm (Laureni et al., 2019). In flocculent sludge, sufficient DO and ammonium supply allowed AOB with more survival advantage to enrich, which provided continuous nitrite to AnAOB (Wang et al., 2018). On the contrary, AnAOB was more prone to form aggregates in biofilms which was equivalent to prolonging the sludge retention time (SRT), providing an effective strategy for enriching slow-growing AnAOB (Zhang et al., 2022). Furthermore, the high mass transfer resistance of biofilm could be considered a reliable barrier to providing better protection for AnAOB against high DO and toxic compound interference (Zheng et al., 2016).

Although AnAOB preferred to be enriched in biofilms, a small fraction of AnAOB (mainly *C. Anammoxoglobus* and *C. Kuenenia*) was still detected in the flocculent sludge (Figures 6B,D), which was probably attributed to the differences in substrate affinity among the different genera of AnAOB. Specifically, *C. Kuenenia* possessed higher oxygen tolerance relative to other genera, which may allow it to survive in the flocs (Oshiki et al., 2016). Due to the capacity of *C. Anammoxoglobus* to degrade organic matter (Kartal et al., 2007b), it is reasonable to speculate that the small molecular organics originating from the CtEG wastewater biodegradation process (Figure 2C) could provide available substrates for its growth (Du et al., 2017). Coupled with the mass transfer limitation of organics in biofilms, allows *C. Anammoxoglobus* to survive in the floc sludge. For another, it is understandable that *C. Anammoxoglobus* and *C. Kuenenia* can easily grow on the surface of biofilm sludge based on the above-mentioned reasons. Therefore, a part of the microbial community flocculent sludge was probably contributed by biofilm detachment (Vlaeminck Siegfried et al., 2010; Yang et al., 2019b).

Based on the LEfSe analysis, the other differential microorganisms apart from AOB and AnAOB were also found in two types of morphological sludge. There into, the most representative biomarkers in flocs were *Denitratisoma* and *Thiobacillus* (Figure 5C), which possessed NO_x_^–^ reduction capacity and were usually regarded as denitrifiers (Fahrbach et al., 2006; Deng et al., 2021). The lower DO of this system (< 0.5 mg⋅L^–1^) allowed the above facultative anaerobic denitrifiers to reside in the flocculent sludge, thus acquiring more organics to provide a better habitat for AnAOB (Yang et al., 2019a). Moreover, such community distribution could further improve the total nitrogen removal efficiency of the PN/A system by reducing effluent nitrate (partial denitrification process), which has been confirmed by our previous study (Yao et al., 2021).

### Spatial Heterogeneity in Each Chamber

At present, most studies focused on the overall differences in microbial communities in different periods of reactor operation, while less attention has been given to the variation of key functional bacteria at different locations in a reactor. To better reveal the spatial heterogeneity of microbial communities, this study explored the distribution of key functional microorganisms in different chambers and depths. The results indicated that the abundance of AOB and AnAOB in flocculent sludge were not significantly (*p* > 0.05) different between each chamber (Figure 4), which may be caused by the uniformly mixing of flocs with the help of sludge recycle and micro-aeration in a plug-flow mode IFAS reactor. Conversely, the abundance of AnAOB in biofilm sludge demonstrated significant differences (*p* < 0.05) between each chamber due to the long SRT of biofilm sludge.

The abundance of the AnAOB genus in the biofilm sludge showed a gradual increasing trend in each chamber (except for *C. Brocadia* in the last chamber, Figures 6B,D), which may be related to the continuous improvement of water quality along with the flow direction. In fact, some microorganisms with a strong degradation ability for toxic or refractory organics were detected in this system, such as *Ignavibacterium*, *Limnobacter*, and *Thiobacillus*, which gradually decreased the toxicity of the wastewater and provide a favorable living environment for AnAOB growth (Ma et al., 2015; Zheng et al., 2019; Li et al., 2020).

In addition, the population shift of AnAOB occurred in each chamber of the IFAS reactor (Figure 6D). Concretely, *C. Brocadia* was the advantage AnAOB with a significantly higher abundance than *C. Kuenenia* and *C. Anammoxoglobus* in the first three chambers. The results of competition among AnAOB genera may be related to niche differences due to their respective growth characteristics. First, based on the theory of priority effect, that is, the establishment of species in a community can depend on the order of their arrival (Debray et al., 2022). In this study, the mature anammox biofilms dominated by *C. Brocadia* were used as the inoculation sludge in the IFAS system (Supplementary Figure 1), which made the *C. Brocadia* depletes resources preferentially, thereby inhibiting the establishment of a late arriver (*C. Kuenenia* and *C. Anammoxoglobus*). Second, the maximum specific growth rates of the *C. Brocadia* genus (0.0027–0.0088 h^–1^) were higher than others, which suggested that it appeared to grow faster and more competitive in biofilms (Oshiki et al., 2016). Third, the *C. Brocadia* has the most hydrophobic surface in extracellular polymeric substances (EPS) than other AnAOB and thus exhibits a superior aggregation capability, which can promote the rapid development of microbial aggregates and enhance the retention effect in biofilms (Jia et al., 2017, 2021; Ali et al., 2018).

Furthermore, the abundance of *C. Brocadia* suddenly decreased in the last chamber and shifted to *C. Kuenenia* and *C. Anammoxoglobu*, which may be related to the following reasons. On the one hand, *C. Kuenenia* possessed a stronger ammonia affinity than *C. Brocadia* (Oshiki et al., 2016), which allowed *C. Kuenenia* to survive in a low ammonia environment, such as the last chamber in this study. On the other hand, *C. Anammoxoglobu* showed outstanding small molecule organic degradation ability compared with *C. Brocadia* (Oshiki et al., 2016). Therefore, some heterotrophic bacteria in this system can continuously decompose complex macromolecular organics into small molecules along the flow direction (He et al., 2021), allowing *C. Anammoxoglobu* to compete for more ecological niches using additional substrates, which also confirms the previous findings of the population shift from *C. Brocadia* to *C. Anammoxoglobu* under propionic condition (Kartal et al., 2007b).

### Significance of This Study

In this study, the reasonable distribution of microbial communities in morphology and space was the key to the successful operation of the autotrophic IFAS system. This suggested that the coexistence of AOB and AnAOB with different physiological characteristics inside the single system was the premise to ensure the stable operation of the IFAS reactor. It is well known that the selection of suitable inoculated sludge is crucial for the successful start-up of the anammox process (Chi et al., 2018). Therefore, the results of niche differentiation in this study inspired the targeted selection of microbial communities in practical engineering applications.

Whether designing new reactors or modifying existing A/O or A/A/O to PN/A process, inoculating with mature anammox sludge of specific genera could be considered depending on the reactor type or water quality. This avoided the population shift of dominant anammox resulting from the long-term sludge adaptive domestication, thus greatly saving system start-up time and achieving stable operation. Based on the findings of this study, we recommended that the front area with high ammonia concentration should be artificially inoculated with *C. Brocadia* dominated sludge, whereas the later area with lower ammonia concentration should be employed with *C. Kuenenia* or *C. Anammoxoglobu* dominated sludge as inoculum in plug-flow PN/A reactor.

Regrettably, more detailed experimental evidence between wastewater chemical components and microbial community metabolism was lacking due to the limitations of the experimental conditions *in situ* of the pilot study. Moreover, the identification of anammox species niches remained a challenge. For further explanation of the morphological and spatial heterogeneity of the microorganisms in IFAS reactor, more attention should be paid to future studies to (1) monitor the changes in the composition and content of toxic or refractory organics from real wastewater; (2) conduct further laboratory studies to explore the effects of different substrate types and concentrations on the niche differentiation among AnAOB species; and (3) employ appropriate anammox inoculated sludge to verify the start-up effect of the PN/A system.

## Conclusion

In this study, the PN/A process was successfully implemented in the pilot-scale IFAS system to treat CtEG wastewater, and the morphological and spatial heterogeneity of microbial community was investigated, with the key conclusions including:

• The IFAS reactor combined different morphologies of sludge (biofilm and floc) to provide an ideal habitat for AnAOB and AOB, respectively;
• Microenvironmental changes caused by the gradient distribution of substrates may be important factors influencing the dominant AnAOB population shift;
• The revelation of spatial heterogeneity provided supporting information for the targeted cultivation and selection of microbial communities in future engineering applications.

## Data Availability Statement

The datasets presented in this study can be found in online repositories. The names of the repository/repositories and accession number(s) can be found in the article/Supplementary Material.

## Author Contributions

FJ: writing—review and editing and funding acquisition. JC: investigation and visualization. XZ: writing—original draft. CL: data curation. YL: methodology and investigation. JM and AY: investigation. HY: conceptualization, supervision, and funding acquisition. All authors contributed to the article and approved the submitted version.

## Conflict of Interest

The authors declare that the research was conducted in the absence of any commercial or financial relationships that could be construed as a potential conflict of interest.

## Publisher’s Note

All claims expressed in this article are solely those of the authors and do not necessarily represent those of their affiliated organizations, or those of the publisher, the editors and the reviewers. Any product that may be evaluated in this article, or claim that may be made by its manufacturer, is not guaranteed or endorsed by the publisher.

## Footnotes

1
https://huttenhower.sph.harvard.edu/lefse/
