# Supplementary figures and images for: Morphological and Spatial Heterogeneity of Microbial Communities in Pilot-Scale Autotrophic Integrated Fixed-Film Activated Sludge System Treating Coal to Ethylene Glycol Wastewater
Source: Front Microbiol. 2022 Jun 2;13:927650. doi: 10.3389/fmicb.2022.927650 (PMC9201488; doi:10.3389/fmicb.2022.927650)

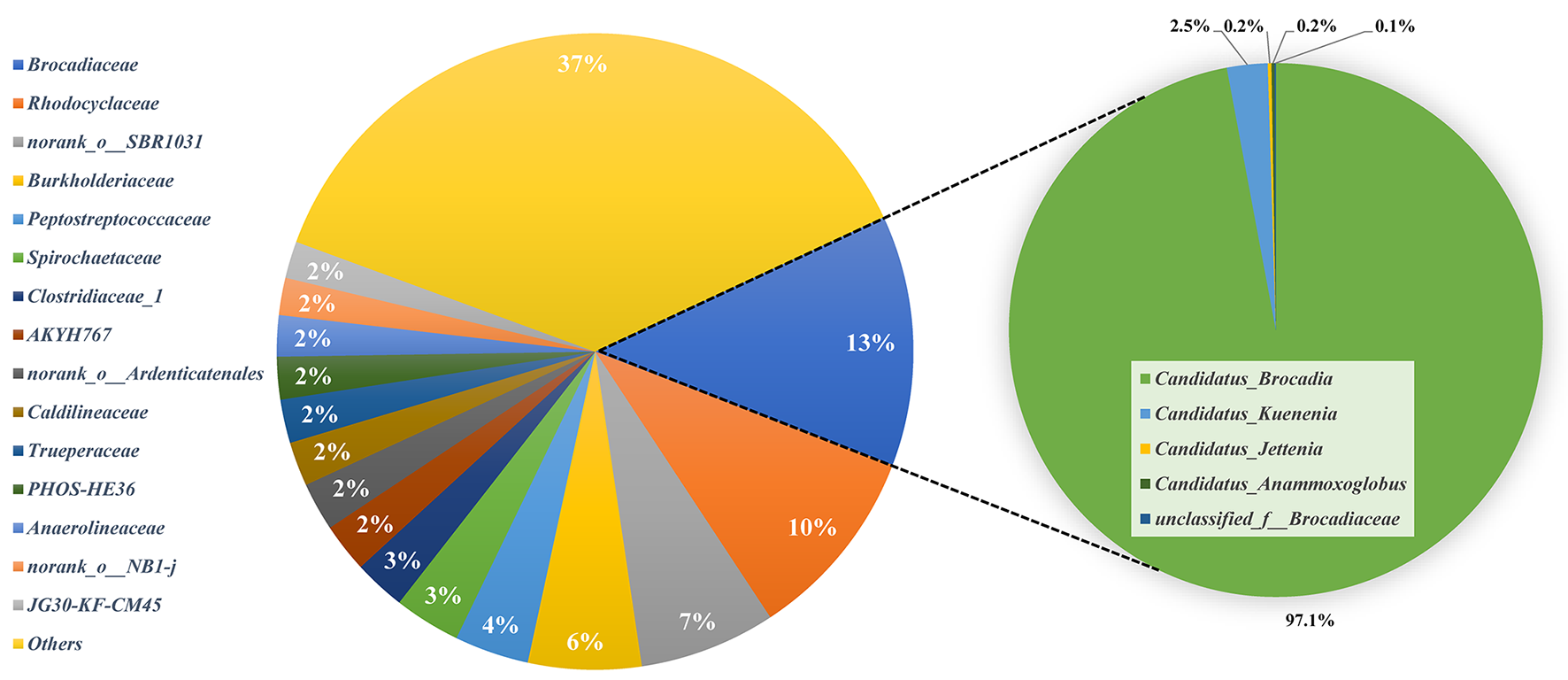

Supplement: Supplementary file 1 [file Image_1.tif]

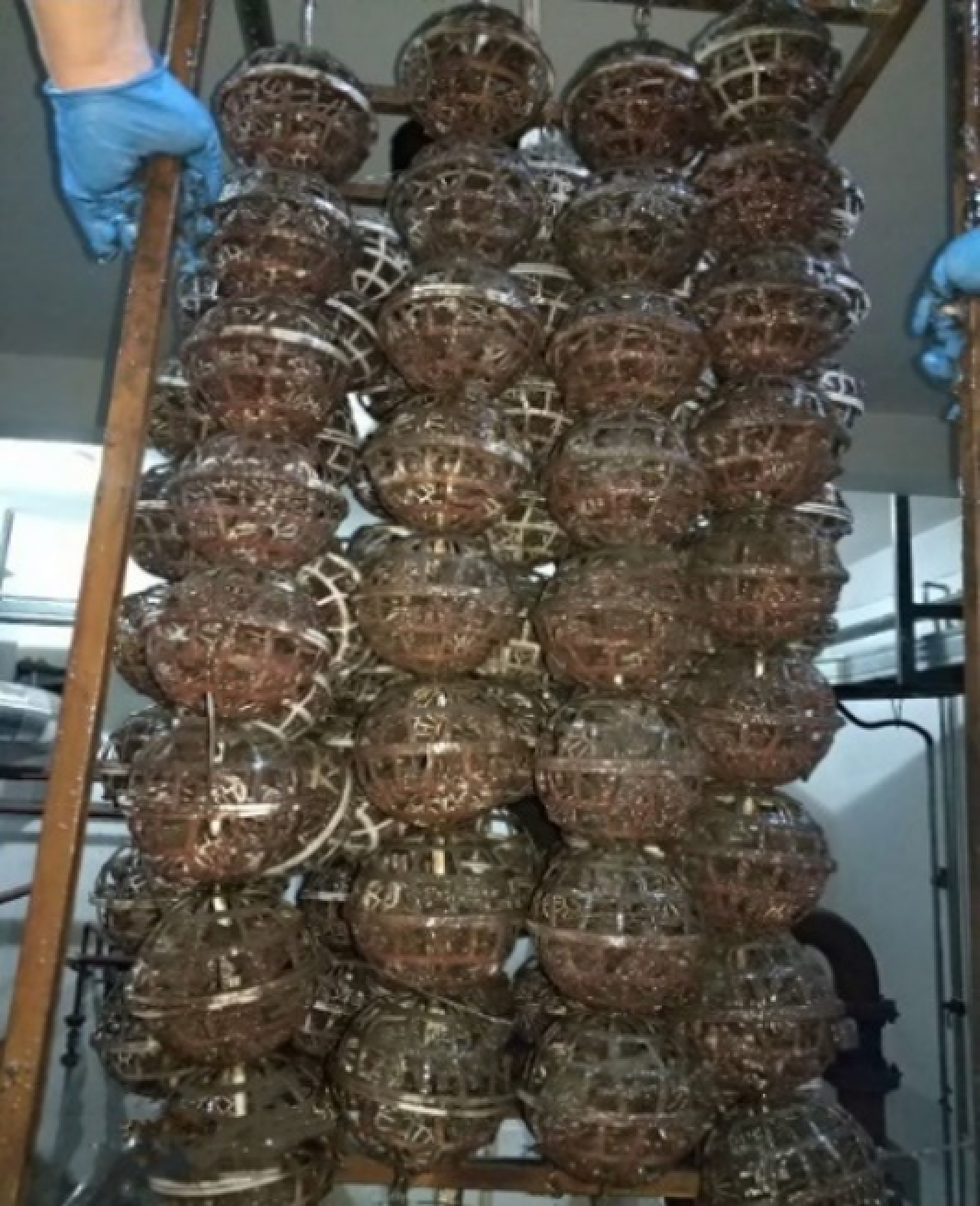

Supplement: Supplementary file 2 [file Image_2.tif]

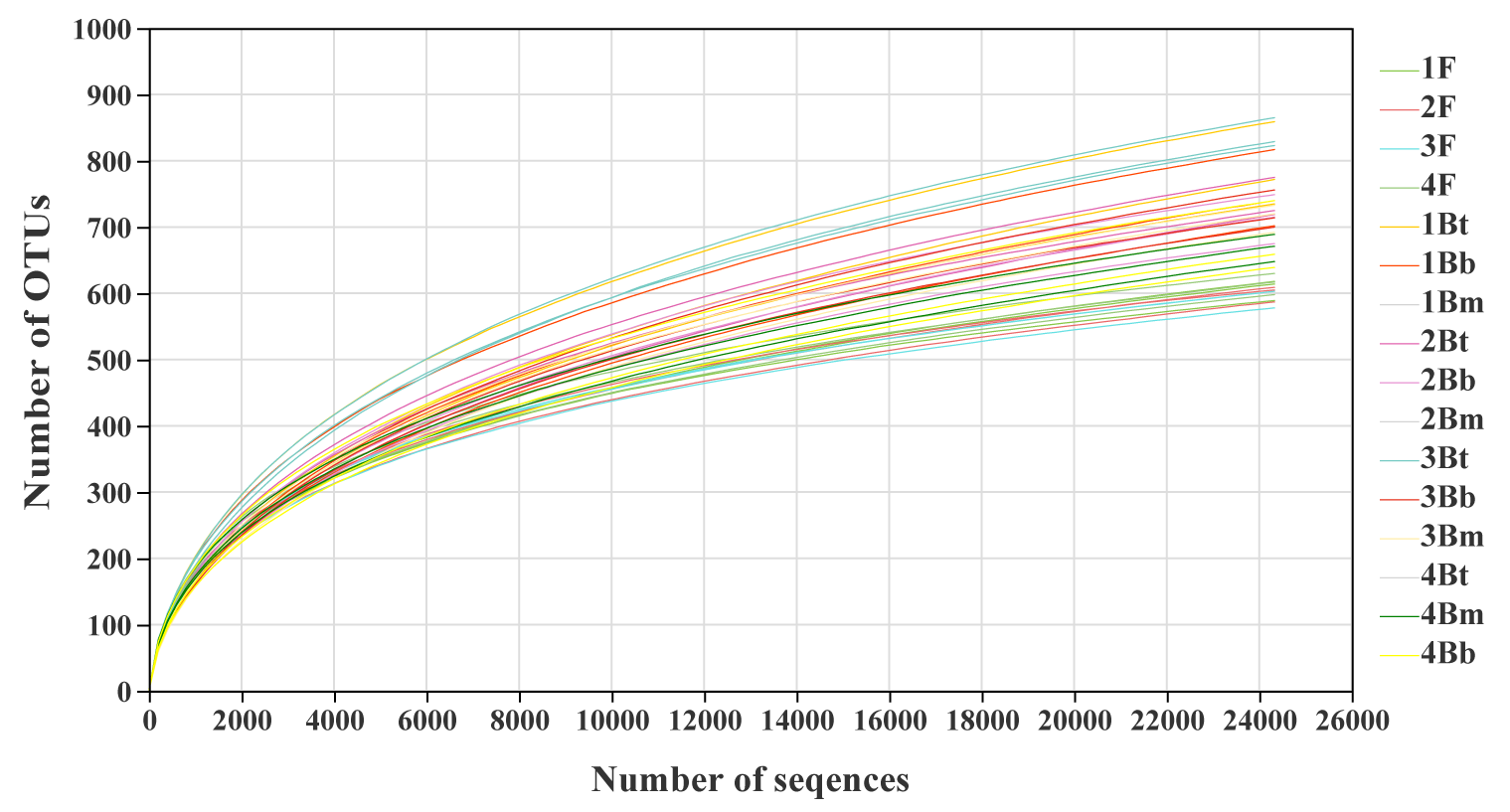

Supplement: Supplementary file 3 [file Image_3.tif]

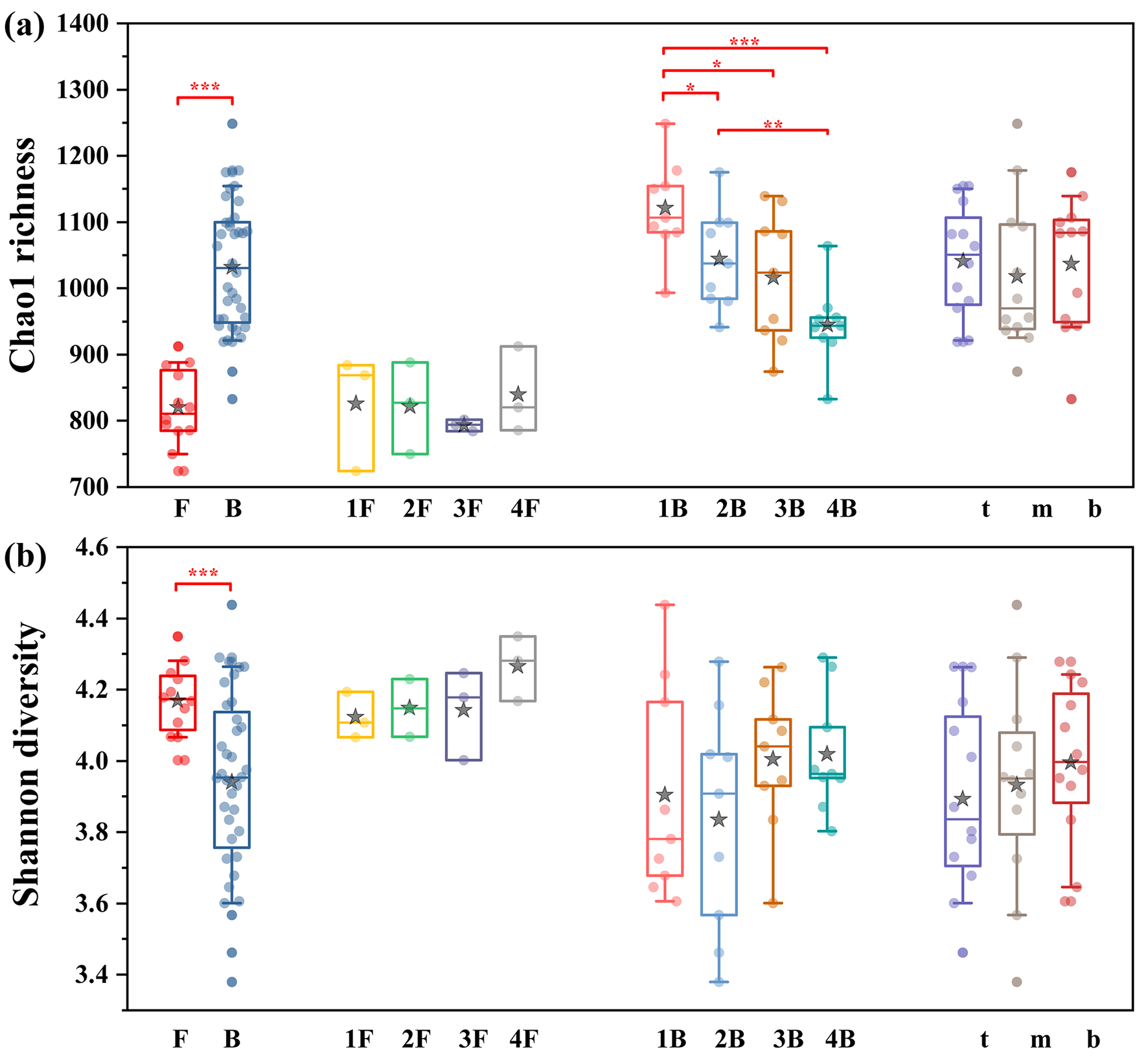

Supplement: Supplementary file 4 [file Image_4.tif]
