# Supplementary material for: Morphological and Spatial Heterogeneity of Microbial Communities in Pilot-Scale Autotrophic Integrated Fixed-Film Activated Sludge System Treating Coal to Ethylene Glycol Wastewater
Source: Front Microbiol. 2022 Jun 2;13:927650. doi: 10.3389/fmicb.2022.927650 (PMC9201488; doi:10.3389/fmicb.2022.927650)
Supplement: Supplementary file 5 [file Table_1.doc]

**Table S1 Richness and diversity of the 48 samples (16×3) based on 0.03 evolutionary distance.**

| **Smaple** | **OTUs** | **Chao1 richness** | **Shannon diversity** | **Good’s coverage (%)** |
| --- | --- | --- | --- | --- |
| 1F | 589 | 724.32 | 4.07 | 0.993 |
|  | 618 | 868.73 | 4.11 | 0.992 |
|  | 614 | 883.88 | 4.19 | 0.992 |
| 1Bt | 772 | 1154.23 | 3.68 | 0.988 |
|  | 859 | 1150.11 | 4.17 | 0.988 |
|  | 735 | 1082.16 | 3.78 | 0.989 |
| 1Bm | 804 | 1093.59 | 3.73 | 0.989 |
|  | 855 | 1248.77 | 3.86 | 0.987 |
|  | 870 | 1177.99 | 4.44 | 0.988 |
| 1Bb | 702 | 993.43 | 3.65 | 0.989 |
|  | 740 | 1084.45 | 3.61 | 0.989 |
|  | 817 | 1106.70 | 4.24 | 0.989 |
| 2F | 587 | 827.51 | 4.07 | 0.992 |
|  | 609 | 888.42 | 4.15 | 0.992 |
|  | 605 | 749.90 | 4.23 | 0.993 |
| 2Bt | 692 | 980.56 | 3.46 | 0.990 |
|  | 720 | 1037.50 | 3.73 | 0.990 |
|  | 733 | 1001.32 | 4.01 | 0.990 |
| 2Bm | 714 | 941.23 | 3.38 | 0.990 |
|  | 700 | 984.29 | 3.57 | 0.990 |
|  | 756 | 1099.13 | 3.91 | 0.989 |
| 2Bb | 865 | 1175.41 | 4.16 | 0.988 |
|  | 829 | 1083.30 | 4.02 | 0.989 |
|  | 823 | 1100.08 | 4.28 | 0.989 |
| 3F | 603 | 784.44 | 4.18 | 0.993 |
|  | 578 | 794.26 | 4.00 | 0.993 |
|  | 603 | 801.37 | 4.25 | 0.993 |
| 3Bt | 593 | 921.45 | 3.60 | 0.990 |
|  | 773 | 1131.35 | 4.26 | 0.988 |
|  | 708 | 1081.62 | 4.08 | 0.990 |
| 3Bm | 715 | 1023.90 | 3.95 | 0.989 |
|  | 749 | 936.69 | 4.04 | 0.990 |
|  | 675 | 874.09 | 4.12 | 0.991 |
| 3Bb | 725 | 954.17 | 3.93 | 0.990 |
|  | 719 | 1085.68 | 3.83 | 0.989 |
|  | 775 | 1139.06 | 4.22 | 0.989 |
| 4F | 617 | 912.50 | 4.28 | 0.992 |
|  | 598 | 785.66 | 4.17 | 0.993 |
|  | 630 | 820.52 | 4.35 | 0.993 |
| 4Bt | 740 | 1063.75 | 4.26 | 0.989 |
|  | 639 | 919.00 | 3.80 | 0.991 |
|  | 659 | 970.58 | 3.87 | 0.990 |
| 4Bm | 648 | 953.37 | 3.96 | 0.991 |
|  | 671 | 925.55 | 3.96 | 0.991 |
|  | 689 | 955.78 | 4.29 | 0.991 |
| 4Bb | 682 | 943.74 | 4.09 | 0.991 |
|  | 673 | 941.13 | 3.95 | 0.991 |
|  | 644 | 833.15 | 3.97 | 0.992 |
